# Infection with a Mouse-Adapted Strain of the 2009 Pandemic Virus Causes a Highly Severe Disease Associated with an Impaired T Cell Response

**DOI:** 10.1371/journal.pone.0138055

**Published:** 2015-09-18

**Authors:** Isabelle Meunier, Olivier Morisseau, Émilie Garneau, Isabelle Marois, Alexandre Cloutier, Martin V. Richter

**Affiliations:** Pulmonary Division, Department of Medicine, Faculty of Medicine and Health Sciences, Université de Sherbrooke and Centre de Recherche du CHUS, Sherbrooke, Québec, Canada; The University of Chicago, UNITED STATES

## Abstract

Despite a relatively low fatality rate, the 2009 H1N1 pandemic virus differed from other seasonal viruses in that it caused mortality and severe pneumonia in the young and middle-aged population (18–59 years old). The mechanisms underlying this increased disease severity are still poorly understood. In this study, a human isolate of the 2009 H1N1 pandemic virus was adapted to the mouse (MAp2009). The pathogenicity of the MAp2009 virus and the host immune responses were evaluated in the mouse model and compared to the laboratory H1N1 strain A/Puerto Rico/8/1934 (PR8). The MAp2009 virus reached consistently higher titers in the lungs over 14 days compared to the PR8 virus, and caused severe disease associated with high morbidity and 85% mortality rate, contrasting with the 0% death rate in the PR8 group. During the early phase of infection, both viruses induced similar pathology in the lungs. However, MAp2009-induced lung inflammation was sustained until the end of the study (day 14), while there was no sign of inflammation in the PR8-infected group by day 10. Furthermore, at day 3 post-infection, MAp2009 induced up to 10- to 40-fold more cytokine and chemokine gene expression, respectively. More importantly, the numbers of CD4^+^ T cells and virus-specific CD8^+^ T cells were significantly lower in the lungs of MAp2009-infected mice compared to PR8-infected mice. Interestingly, there was no difference in the number of dendritic cells in the lung and in the draining lymph node. Moreover, mice infected with PR8 or MAp2009 had similar numbers of CCR5 and CXCR3-expressing T cells, suggesting that the impaired T cell response was not due to a lack of chemokine responsiveness or priming of T cells. This study demonstrates that a mouse-adapted virus from an isolate of the 2009 pandemic virus interferes with the adaptive immune response leading to a more severe disease.

## Introduction

Influenza A viruses (IAV) are responsible for yearly epidemics and sporadic pandemics. Because of the segmented structure of the viral genome, exchange of genetic material between viruses is possible, thus allowing the generation of new viral strains that may have high pandemic potential [1]. Furthermore, IAV viruses that have acquired the ability to cross the species barrier and to infect humans are often associated with high virulence. For instance, the 1918 “Spanish Flu” that caused between 20–50 million deaths worldwide, is thought to originate from an avian-to-human antigenic shift that acquired the capacity to infect human [2,3,4]. Moreover, human infection by the highly pathogenic H5N1 viruses is associated with the development of acute respiratory distress syndrome and respiratory failure, leading to a lethal outcome in up to 60% of individuals [5]. In 2009, a virus resulting from the reassortment of genes originating from human, swine, and avian viruses acquired the ability to infect humans and spread in the population causing the first pandemic of the 21^st^ century (A(H1N1)pdm09) [6,7]. While the overall death rate was comparable to seasonal IAV, the pandemic virus differed from seasonal viruses in that up to a third of the severely ill patients were young to middle-aged individuals, rather than the very young or elderly populations. In addition, the main cause of death from A(H1N1)pdm09 was viral pneumonia rather than being associated with bacterial infection [8,9,10].

Factors contributing to pathogenesis and disease severity are still poorly understood but certainly comprise virulence factors particular to each IAV strain and the ability of the host to respond to the infection. Many viral proteins have been shown to contribute to IAV virulence. Indeed, mutations in the hemagglutinin (HA) affect tissue tropism and host cellular range, while mutations in viral polymerases, especially PB2, are associated with mammalian adaptation [11,12,13,14,15,16]. Moreover, mutations in viral neuraminidase (NA) promote virulence [17,18,19]. PB1-F2, a protein encoded in the +1 reading frame of the PB1 segment, also contributes to virulence by inducing apoptosis and increasing the severity of secondary bacterial infection [20,21]. Finally, NS1 interferes with the innate immune response [22,23,24]. Interestingly, the 2009 pandemic virus (A(H1N1)pdm09) does not possess most of these virulence factors [23,25,26,27,28].

The host immune response to A(H1N1)pdm09 is still elusive. Fatal human cases were associated with extensive diffuse alveolar damage and viral replication mainly in the lung parenchyma [29,30,31]. These patients also exhibited a remarkable elevation of IL-1RA, IL-6, IL-8, TNF-α, MCP-1, MIP-1β, and IP-10 in the lungs, which correlated with the peak of viral replication [9,32,33]. Interestingly, some studies have shown that severely ill patients had a deficiency in the genes and cells involved in adaptive immunity, such as in antigen presentation, B-cell development, and T-helper cell differentiation [8,34,35]. Furthermore, studies in mice, macaques, and ferrets confirmed that different isolates of the A(H1N1)pdm09 virus causes up-regulation of many pro-inflammatory cytokines, increased cellular infiltration in the airways, and are associated with increased morbidity and death [31,36,37,38,39,40]. However, very few studies have investigated the impact of the virus on the adaptive immune response. In this study, we sought to investigate how infection by the A(H1N1)pdm09 virus influences the immune response. A mouse-adapted A(H1N1)pdm09 virus was generated by consecutive passages in mouse lungs, yielding the MAp2009 virus. We compared the pathogenicity, histopathological changes and viral loads of MAp2009 to the conventional PR8 virus. The cytokine response and the innate and adaptive cellular immune responses were then quantified. We observed that both viruses replicated with different efficiency in mouse lungs and triggered a distinct innate and adaptive immune response.

## Materials and Methods

### Cells and viruses

MDCK cells (ATCC CCL-34) were maintained in EMEM (Wisent) supplemented with 10% FBS, antibiotics (penicillin 100 U/ml and streptomycin 100 mg/ml), 5% sodium pyruvate, 2mM L-glutamine, and 5% non-essential amino acids (Wisent). The original stock of the mouse-adapted A/H1N1/Puerto Rico/8/1934 (PR8) was provided by Dr David Topham (University of Rochester Medical Centre, New York Influenza Centre of Excellence, Rochester, NY, USA) and amplified in 10-day-old embryonated hen’s eggs as described previously [41]. The human isolate H1N1 A/California-like/2009 was isolated in Montreal, Canada, during the 2009 pandemic and obtained from Dr. Hugues Charest of the Laboratoire de santé publique du Québec. The virus was adapted to mouse by 8 consecutive passages as previously described [42], generating MAp2009, and was further amplified in MDCK cells.

### Animal experiments

All experiments were approved by the Institutional Animal Ethics Committee of the Faculté de Médecine et des Sciences de la Santé of the Université de Sherbrooke. Female C57BL/6 mice (18–20 g) were purchased from Charles River Laboratories (Portage, MO, USA) and housed in specific pathogen-free conditions at the Institutional Animal Care Facility. The animals were anesthetized by intraperitoneal (i.p.) administration of 240 mg/kg of Avertin (2.2.2–tribromoethanol, Sigma-Aldrich) and infected intranasally (i.n.) with 10 plaque-forming units (PFU) of PR8 or MAp2009 in 30 μl of PBS. Mouse weight was evaluated daily to monitor morbidity. A weight loss greater than 35% of the original weight was considered as the critical limit of the experiments and mice were euthanized according to the Canadian Council on Animal Care.

#### Cell isolation

At the indicated time points, animals were euthanized by i.p. injection of 720 mg/kg of Avertin and exsanguinated by cardiac puncture or by sectioning the inferior vena cava. Lungs were purged of blood by injecting 10 ml of PBS in the right ventricle of the heart. The spleen was removed and placed in EMEM. For bronchoalveolar lavage (BAL), an incision was made below the jaw and the trachea was canulated. Three lavages were performed with 1 ml of EMEM. Whole lungs were flushed by injection of 10 ml of PBS in the right ventricle of the heart and placed in EMEM. Finally, the mediastinal lymph node (MLN) was removed and placed in EMEM. Spleens and MLNs were homogenized in Dounce homogenizers in EMEM. Cell suspensions were filtered through a 90-μm nylon mesh and centrifuged for 5 min at 400 x *g*. Spleen cells were resuspended in 1 ml of EMEM and red blood cells were lysed by addition of 2 ml of buffered ammonium chloride solution (Gey’s solution) for 2 min. The lysis was stopped by adding EMEM, cells were centrifuged, resuspended in 8 ml of EMEM and filtered through a 70-μm nylon mesh. Lungs were homogenized by gently rubbing organs over a 200-μm gauge wire mesh and filtered through a 90-μm nylon mesh. Cell suspensions were centrifuged and supernatants were stored at -80°C for further titration. Lung lymphocytes were isolated by a Histopaque 1083 (Sigma-Aldrich) gradient centrifugation at 800 x *g* for 30 min. Cells were washed once and resuspended in EMEM and filtered through a 70-μm nylon mesh. Finally, BALs were centrifuged and cell pellets were resuspended in EMEM and filtered on a 70-μm nylon mesh. Cell counts of all cell suspensions were determined by Trypan blue exclusion.

#### Flow cytometry staining of innate immune cells

Fc receptors on isolated cells were blocked with a 1:300 dilution of anti-CD16/32 (eBioscience) for 30 min at 4°C, washed with PBS-BSA 0.2%, and labeled with a 1:100 dilution of anti-NK1.1 (BV650; BioLegend), 1:300 of anti-F4/80 (FITC or PerCPCy5.5; BioLegend), 1:400 of anti-CD3ε (BV421; BD Bioscience), anti-γδ TCR (APC; eBioscience), and anti-B220 (V500; BD Bioscience), 1:600 of anti-Gr1 (APC-Cy7; BD Biosciences), 1:800 of anti-CD11b (PE; BD Bioscience or FITC; eBioscience) and anti-CD11c (PE-Cy7; eBioscience), and 1:1600 anti-MHCII (Alexa Fluor700 or APC; BioLegend). After 30 min at 4°C, cells were washed twice and fixed in 2% paraforlmadehyde (PFA; Alfa Aesar) diluted in PBS-BSA 0.2% for 15 min at 4°C. Cells were washed, resuspended in PBS-BSA 0.2%, and analyzed by flow cytometry. Samples were collected with FACSDiva Software using a BD FACS Aria III and post-acquisition analyses were performed using FlowJo (Tree Star, Ashland, OR, USA).

#### Cell stimulation and flow cytometry staining of adaptive immune cells

Analysis of the T cell response was performed as described previously [43]. Briefly, 10^6^ cells were seeded in a 96-well plate and stimulated with 1 μM of NP and PA peptide epitopes specific to the virus in presence of 1μl/ml of BD Golgi Plug (Brefeldin A, BD Biosciences) in EMEM 10% FBS. The influenza viral epitopes NP_366-374_ ASNENMETM and PA_227-233_ SSLENFRAYV were used for PR8 and NP_366-374_ ASNENVETM and PA_227-233_ PSLENFRAYV for MAp2009 [44]. After 6 h at 37°C, cells were washed, blocked and labeled with a 1:400 dilution of anti-CD8 (PerCP-Cy5.5, V500; BD Bioscience or APC-eFluor780; eBioscience), anti-CD3ε (APC or BV421; BD Bioscience), anti-CD4 (V450; BD Bioscience or APC; eBioscience), anti-CD62L (APC-Cy7; BD Bioscience or FITC; eBioscience), and a 1:800 dilution of anti-CD44 (PE-Cy7; BD Bioscience). Intracellular staining was performed using 100 μl of BD Perm/Wash buffer (BD Bioscience) for 20 min at 4°C. After centrifugation for 5 min at 400 x *g*, cells were stained for IFN-γ (anti-IFN-γ PE; BD Bioscience) and TNF-α (anti- TNF-α FITC or Alexa Fluor700; BD Biosciences) at a 1:200 dilution for 30 min at 4°C. Cells were washed with BD Perm/Wash buffer and resuspended in PBS-BSA 0.2% and analyzed by flow cytometry. All samples were collected with FACSDiva Software using a BD FACS Canto and post-acquisition analyses were performed using FlowJo (Tree Star, Ashland, OR, USA).

#### Chemokine receptor expression on CD8^+^ T cells

Fc receptors on isolated cells were blocked as described above. Cells were washed and labeled with a 1:200 dilution of anti-CCR5 (PE; BioLegend) and anti-CXCR3 (APC; BioLegend) or isotype controls (PE Armenian Hamster IgG (BioLegend) or APC Armenian Hamster IgG (BioLegend), respectively) in combination with a 1:400 dilution of anti-CD8 (PerCP-Cy5.5; BD Bioscience), anti-CD4 (Fitc; eBioscience), anti-CD62L (APC-Cy7; BD Bioscience), and a 1:800 dilution of anti-CD44 (PE-Cy7; BD Bioscience) for 30 min at 4°C. After two washes, cells were fixed as previously described and samples were collected with FACSDiva Software using a BD FACS Canto and post-acquisition analyses were performed using FlowJo.

#### Identification of dendritic cell subsets

Fc receptors on isolated cells were blocked with a 1:300 dilution of anti-CD16/32 (eBioscience) for 30 min at 4°C, washed with PBS-BSA 0.2%, and labeled with a 1:400 dilution of Ly-6C (APC-Cy7; BD Bioscience), CD11c (PE-Cy7; BD Bioscience), B220 (V500; BD Bioscience), CD103 (PerCP-Cy5.5; BioLegend), a 1:1600 dilution of MHCII (APC; BioLegend), and a 1:4000 dilution of CD11b (PE; BD Bioscience) for 30 min at 4°C. Cells were washed twice in PBS-BSA 0.5% and fixed in 2% PFA diluted in PBS-BSA 0.2% for 15 min at 4°C. Cells were washed, resuspended in PBS-BSA 0.2%, and analyzed by flow cytometry. Samples were collected using a BD FACS Aria III with FACSDiva software and post-acquisition analyses were performed using FlowJo.

#### Virus titration

Virus titers in the lungs were determined by viral plaque assay as previously described [43]. Confluent MDCK cells seeded in a 24-well plate were washed twice with PBS and infected with 300 μl of 10-fold dilutions of lung homogenates. After 1h, supernatants were aspirated, cells were washed twice with PBS, and 750 μl of Avicel 1.8% (Avicel 3.6%, kindly provided by FMC BioPolymer, Philadelphia, PA, USA, diluted 1:1 in 2X EMEM) containing 1 μg/ml of TPCK-treated trypsin was added. After 48h, medium was aspirated, cells were washed twice with PBS and fixed for 20 min at 4°C with Carnoy fixative (methanol:acetic acid, 3:1). Viral plaques were then revealed by staining with 1% crystal violet solution in 20% methanol for 5–10 min. Numbers of plaques were counted and the viral titer was determined according to the dilution and expressed as PFU.

#### Evaluation of lung pathology

Animals were sacrificed at days 2, 4, 8, and 10 post-infection and lungs were gently flushed by injection of 10 ml of PBS in the right ventricle of the heart. Lungs were fixed in 10% neutral buffered formalin, paraffin-embedded, cut in 5-μm-thick section, and stained with hematoxylin and eosin (H&E). Slides were evaluated in a blinded fashion and images were taken using an Axioskop2 microscope (Zeiss) and analysed with the ImagePro software (Media Cybernetics, Warrendale, PA).

#### RNA extraction and quantification of cytokines by semi-quantitative PCR

Lung homogenates (200 μl) were dissolved in 1 ml of TRIzol reagent (Invitrogen) and RNA was extracted following to the manufacturer’s instructions. RNA (1μg) was then reverse-transcribed with random decamers (Ambion, Austin, TX, USA) using the OmniScript RT kit (Qiagen) according the manufacturer’s protocol. Cytokine gene expression and the housekeeping gene HPRT were quantified by qPCR using the Quantitect SYBR Green PCR kit (Qiagen) in the Rotor-Gene 6000 real-time PCR system (Corbett, Qiagen) with the following protocol: denaturation at 95°C for 5 min, followed by 50 cycles of 95°C for 25 sec, 55.1°C for 35 sec, and 72°C for 35 sec, and a melting curve analysis to confirm sample specificity. The fold change ratios between infected animals to uninfected controls were calculated and normalized to HPRT using the ΔΔCT method [45].

#### Evaluation of the humoral immune response

Sera were collected from the facial vein of mice at days 4, 7, 14, and 21 post-infection (p.i.). To determine influenza IgG titers, immunoperoxidase monolayer assays (IPMA) were performed according to a previously published protocol [46]. Briefly, confluent MDCK cells seeded in a 96-well plate were infected with 100 PFU of PR8 or MAp2009 for two days. Plates were washed twice with PBS diluted 1:3 in water, dried, and fixed overnight at 65°C. Serial dilutions of mouse sera were added to the plates in quadruplicate for 1h. Plates were washed twice with PBS and a 1:500 dilution of a peroxidase-labeled anti-mouse IgG (Jackson ImmunoResearch) was added for 30 min. After washing twice with PBS, virus specific staining was visualized by the addition of 280 mg/ml of 3-amino-9-ethylcarbazole (Sigma) and 0.01% hydrogen peroxide in 50 mM acetate buffer (pH 5.0). Antibody titer is expressed as reciprocals of the highest dilution at which viral antigen was detected by light microscopy.

The influenza hemagglutination-inhibition antibody titer assays (HAI) were performed as previously described [41]. Briefly, sera collected at day 21 were decomplemented by heating at 65°C for 30 min and incubated with chicken erythrocytes (Lampire Biological Laboratories) to remove non-specific agglutination activity. Next, serial dilutions of sera were incubated with 4 HAI units of PR8 or MAp2009 prior to incubation with erythrocytes. The HAI titer was determined as the reciprocal of the highest dilution completely inhibiting erythrocyte agglutination.

### Statistical analyses

The statistical analyses were performed using GraphPad Prism 5.0. *P* values were calculated by individually comparing the different groups using an unpaired Mann-Whitney test.

## Results

### MAp2009 infection is associated with increased morbidity, decreased survival, and higher viral loads

**Fig 1:**
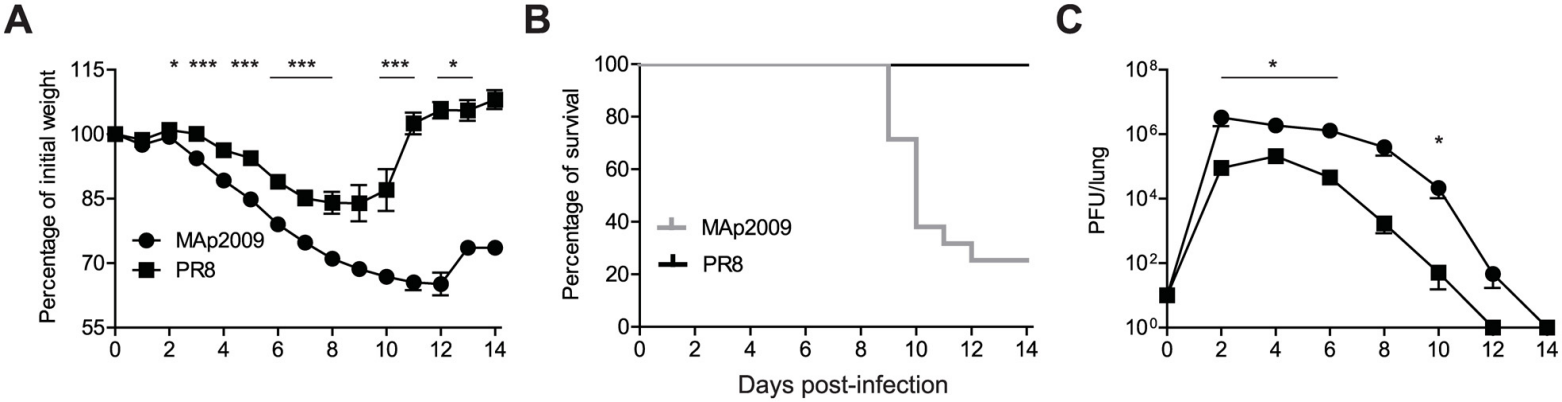
MAp2009 virus causes a more severe infection with more morbidity, less survival, and higher lung viral loads. Mice were infected i.n. with 10 PFU of MAp2009 or PR8. (**A**) Body weight was monitored daily for a 14-day period. Data represent a percentage of the initial weight. (**B**) Survival rates. (**C**) Lung viral titers. Mice were euthanized at the indicated time points and lung viral titers were determined by viral plaque assay. Data were analysed using a Mann-Whitney test (*P< 0.05, **P< 0.01, ***P< 0.001,) and each value represent the mean of at least 4 mice.

To assess and compare the virulence of MAp2009, C57BL/6 mice were infected with 10 PFU of MAp2009 or PR8 viruses and morbidity, as assessed by weight loss, and mortality were monitored for 14 days. Animals infected with the PR8 virus experienced transient weight loss that started at day 4 p.i. and attained up to 83% of initial weight. All animals regained their initial weight by day 11 (Fig 1A). Strikingly, mice infected with the MAp2009 virus presented a more severe disease, as assessed by weight loss starting at day 3 p.i., reaching up to 65% by day 12 p.i. (some animals had to be euthanized for ethical reasons). Furthermore, infection with the PR8 virus did not cause any mortality while MAp2009 was associated with an overall 85% mortality rate, starting at day 9 p.i. (Fig 1B). Since increased mortality is often associated with high viral titers, we next assessed the lung viral titers in MAp2009- and PR8-infected mice. In MAp2009-infected animals, we observed a rapid increase in viral load that peaked at day 2 p.i. and stayed roughly stable until day 6 (Fig 1C). In PR8-infected mice, viral titers rose rapidly as well but peaked at day 4 p.i. and started to decrease thereafter. Interestingly, MAp2009 titers remained 10 to 100-fold higher throughout the course of the experiment and lung viral titers were sustained until day 14 p.i., while the PR8 virus was cleared from the lungs by day 12. This difference could not be attributed to a growth advantage as both viruses had very similar growth curves in MDCK cells (data not shown). Thus, these results suggest that inefficient control of viral replication and delayed viral clearance in the MAp2009-infected group is associated with increased morbidity and mortality.

**Fig 2:**
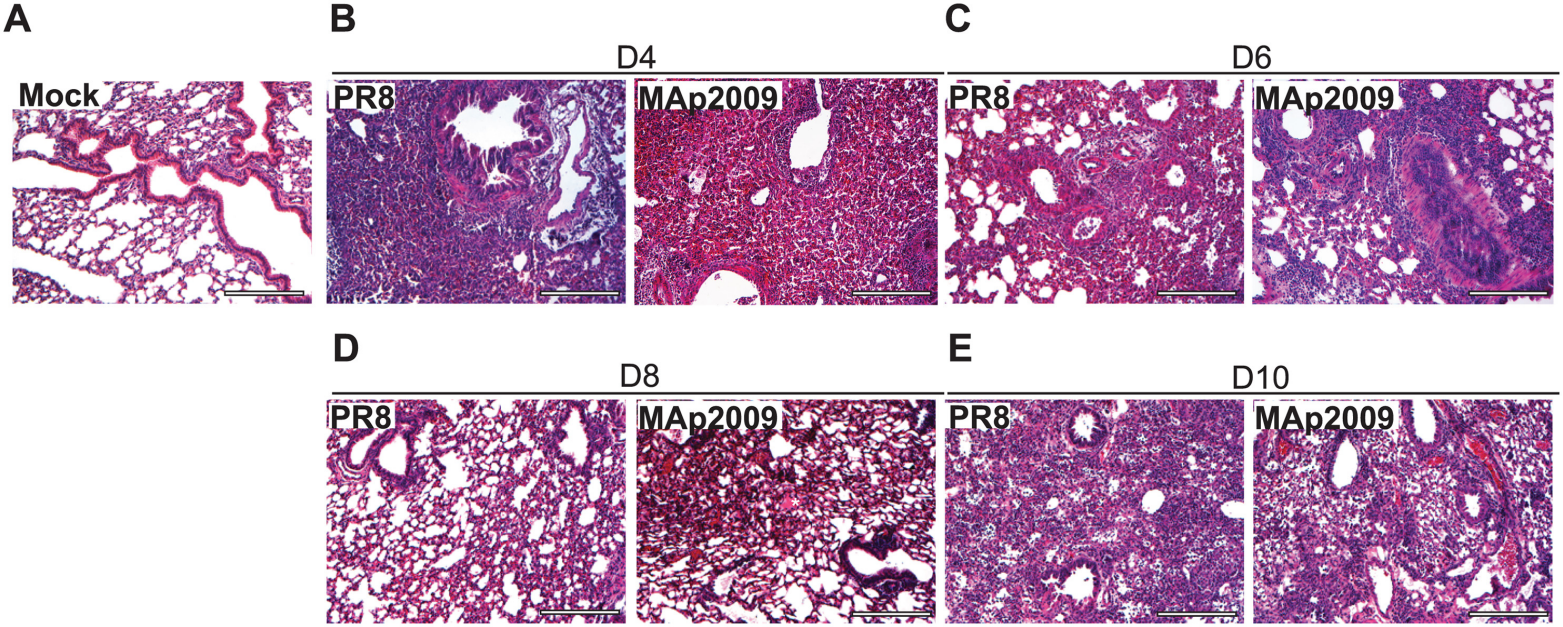
Pathological changes are observed after infection with the MAp2009 virus. Histopathological examination of H&E-stained slides of the lungs of MAp2009 and PR8-infected animals at different time post-infection. (**A**) Mock-infected control. Lungs of mice infected with PR8 or MAp2009 virus, respectively, at day 4 (**B**), 6 (**C**), 8 (**D**), and 10 (**E**) post-infection. Mice were euthanized at the indicated time points and whole lungs were flushed with 10 ml of PBS, formalin-fixed, paraffin-embedded, and H&E stained. Images were taken at a 10X magnification. Scale bar represents 250 μm.

To further characterize the pathology caused by MAp2009, we compared the extent and the nature of lung damage to PR8 at specific time p.i. Infection by MAp2009 and PR8 caused rapid changes in the lung parenchyma. At day 2 p.i., infection by both viruses were associated with cellular infiltration, loss of bronchiolar epithelium, occasional thickening of the alveolar walls and sporadic edematous areas (data not shown). At day 4, when viral titers were maximal, cellular infiltration was dramatically increased and alveolar walls remained thickened, suggesting the recruitment of innate cells as well as the beginning of the inflammatory response. Furthermore, many bronchi lost their epithelial cell layer and large consolidated areas in the lungs were observed in both groups (Fig 2A and 2B). After 6 days p.i., while viral titers began to decrease, bronchiolar damage was still present and cellular infiltration was associated with the consolidated areas (Fig 2C). However, PR8-infected animals had larger and more frequent consolidated areas than the MAp2009 group. In both groups, alveolar thickening was still present in most of the lungs, but some regions were less affected. Finally, MAp2009-infected animals had more and larger edematous areas compared to the animals infected with the PR8 virus. Cellular infiltration as well as bronchiolar damage became less severe at day 8 p.i. for both groups (Fig 2D). However, MAp2009-infected animals still presented more cellular infiltrates, consolidated areas, and deepithelialized bronchi, than mice infected with PR8. Finally, at day 10, the numbers of damaged bronchi were reduced in both groups, correlating with the decrease in viral loads (Fig 2E). However, cellular infiltrates were still present and alveolar walls remained thickened. For both groups, damage seemed to be restricted to specific lobes rather than being spread throughout the lungs. Therefore, the extent of lung pathology seems to be associated with viral loads.

### Morbidity and high viral loads could not be attributed to a difference in the accumulation of innate immune cells

**Fig 3:**
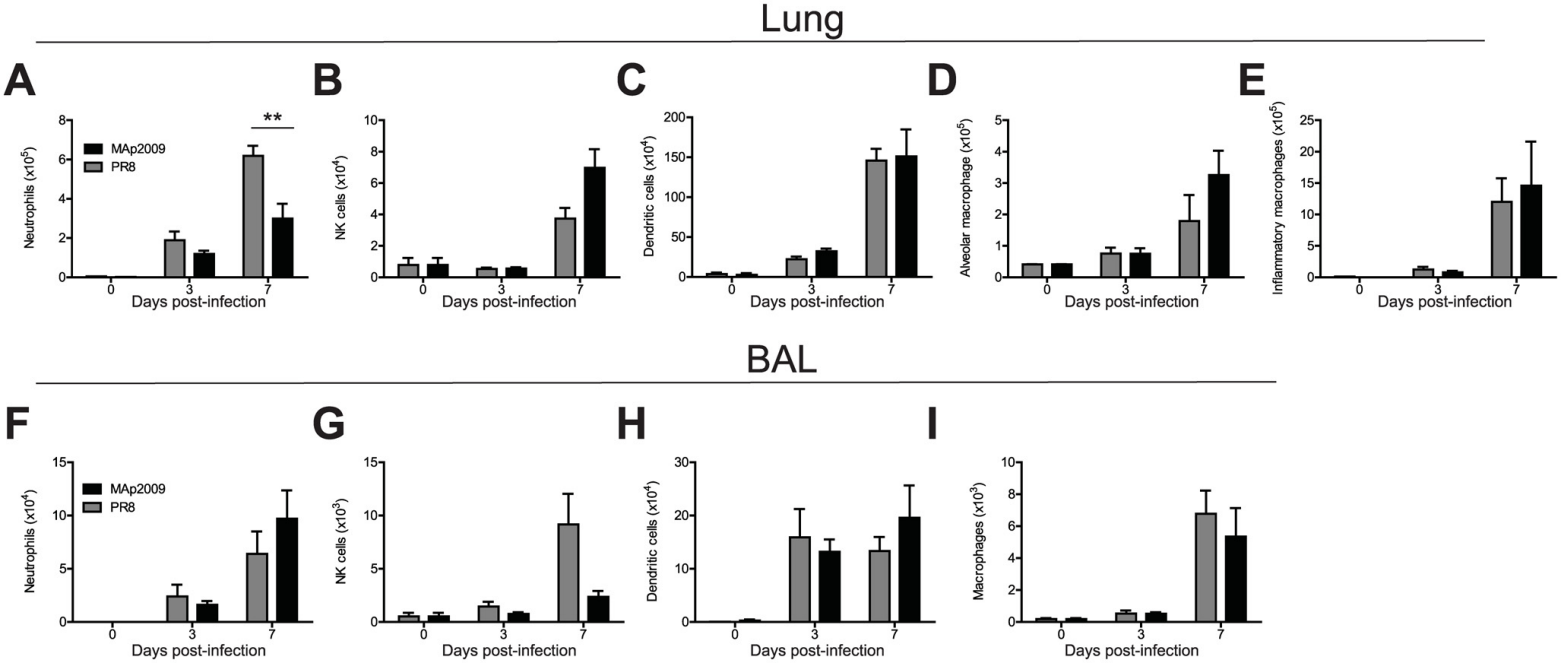
Infection with the MAp2009 virus has little effect on the accumulation of innate immune cells. Characterization of innate immune cell populations by flow cytometric analyses in the lungs (**A-E**) and in the bronchoalveolar lavages (BAL) (**F-I**). Mice were infected with 10 PFU of PR8 or MAp2009, lungs and BAL were harvested at the indicated time points, and cells were analyzed by flow cytometry. Neutrophils were identified as CD11c^lo^F4/80^-^CD11b^hi^Gr-1^+^, NK cells as CD3^-^NK1.1^+^, DCs as CD11c^hi^CD11b^hi^Gr-1^hi^, and macrophages as F4/80^+^CD11c^lo^CD11b^hi^Gr-1^-^. In the lungs, inflammatory macrophages were distinguished from alveolar macrophages by the high expression of CD11b. Data were analysed by an unpaired Mann-Whitney test (*P< 0.05, **P< 0.01) and represent the mean of at least 5 animals in experiments performed in triplicate.

The innate immune response following IAV infection is crucial for the initial control of viral replication, to prevent its dissemination, and to orchestrate the adaptive immune response [47,48]. To determine whether the difference in the viral loads and morbidity could be attributed to an impaired or exacerbated innate immune response, we quantified the number of innate immune cells present in the airways at day 3 and 7 p.i. by flow cytometry. Infection with both viruses triggered a comparable recruitment of neutrophils, NK cells, dendritic cells (DC), and macrophages as early as day 3 p.i. in the lung parenchyma (Fig 3A–3E). The cellularity increased until day 7 and was equivalent between both groups, except for the number of neutrophils that was significantly higher in the PR8-infected group (Fig 3A). Similarly, in the bronchoalveolar lavages (BAL), the presence of innate immune cells was detected at day 3 p.i. and the infiltration was between 2 to 8-fold greater at day 7 (Fig 3E–3H). Again, no noticeable difference was observed between both groups. These data thus suggest that a difference in the recruitment of innate immune cells is not responsible for the increased morbidity and high viral loads in the MAp2009-infected animals.

### Infection of mice with MAp2009 induces a strong cytokine response early upon infection

**Fig 4:**
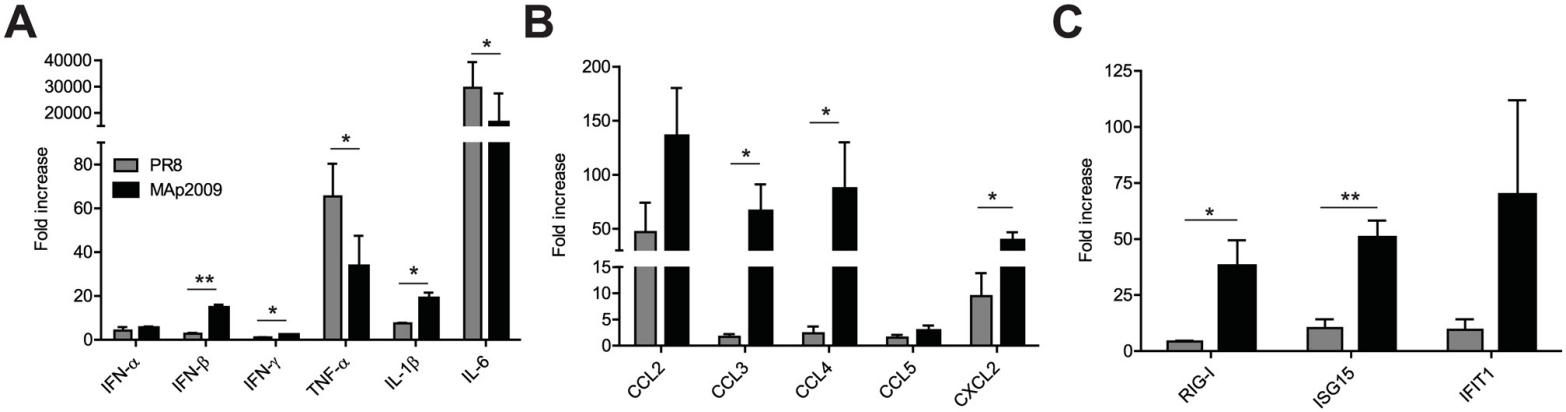
Infection by the PR8 and MAp2009 viruses elicits a distinct cytokine profile. (**A**) Induction of type I and type II IFN and the pro-inflammatory cytokines TNF-α, IL-1β, and IL-6. (**B**) Induction of genes encoding for chemokines. (**C**) Induction of interferon-stimulated gene expression. Mice were infected i.n. with 10 PFU of PR8 or MAp2009. RNA from lung cells was extracted at day 3 p.i., reverse transcribed, and gene expression was quantified by semi-quantitative PCR. Each value represents the mean relative mRNA expression in the lungs of at least 3 mice originating from a minimum of 2 independent experiments. Data were analyzed with an unpaired Mann-Whitney test (*P< 0.05, **P< 0.01).

To investigate if the local cytokine response could contribute to the differences observed in pathogenesis, we next quantified the expression of a panel of cytokines, chemokines, and interferon-stimulated genes (ISG) in the lungs of animals at day 3 p.i., which coincides with the initiation of weight loss (Fig 1A). Infection with both viruses triggered the induction of type I and type II interferons (IFN-α, IFN-β, and IFN-γ) (Fig 4A). However, infection with the MAp2009 virus led to 7-fold more IFN-β and 2-fold more IFN-γ gene expression (*P*<0.05, *P*<0.01, respectively). The expression of the pro-inflammatory cytokines TNF-α, IL-1β, and IL-6 in lung homogenates was next assessed. While PR8-infected animals had about 3-fold more TNF-α and 2-fold more IL-6, mice infected with MAp2009 had 2-fold more IL-1β (*P*<0.05). Furthermore, qPCR analyses revealed that infection with PR8 or MAp2009 triggered the expression of chemokines, with MAp2009 generally inducing a stronger response (Fig 4B). Notably, CCL3 expression was approximately 30-fold higher, CCL4, 40-fold higher and CXCL2, 3-fold higher (*P*<0.05) in MAp2009-infected animals. Lastly, we also analysed the expression of ISG in the lungs of infected animals. In accordance with the stronger IFN-β induction in MAp2009-infected mice, expression of RIG-I and ISG15 was 8- and 5-fold higher than in the lungs mice infected with the PR8 virus, respectively (*P*<0.05) (Fig 4C). Expression of IFIT1 was also 8-fold higher in the MAp2009-infected group, but the difference between groups was not statistically significant.

### Infection with the MAp2009 virus leads to an impaired T cell response while having little impact on antibody production

**Fig 5:**
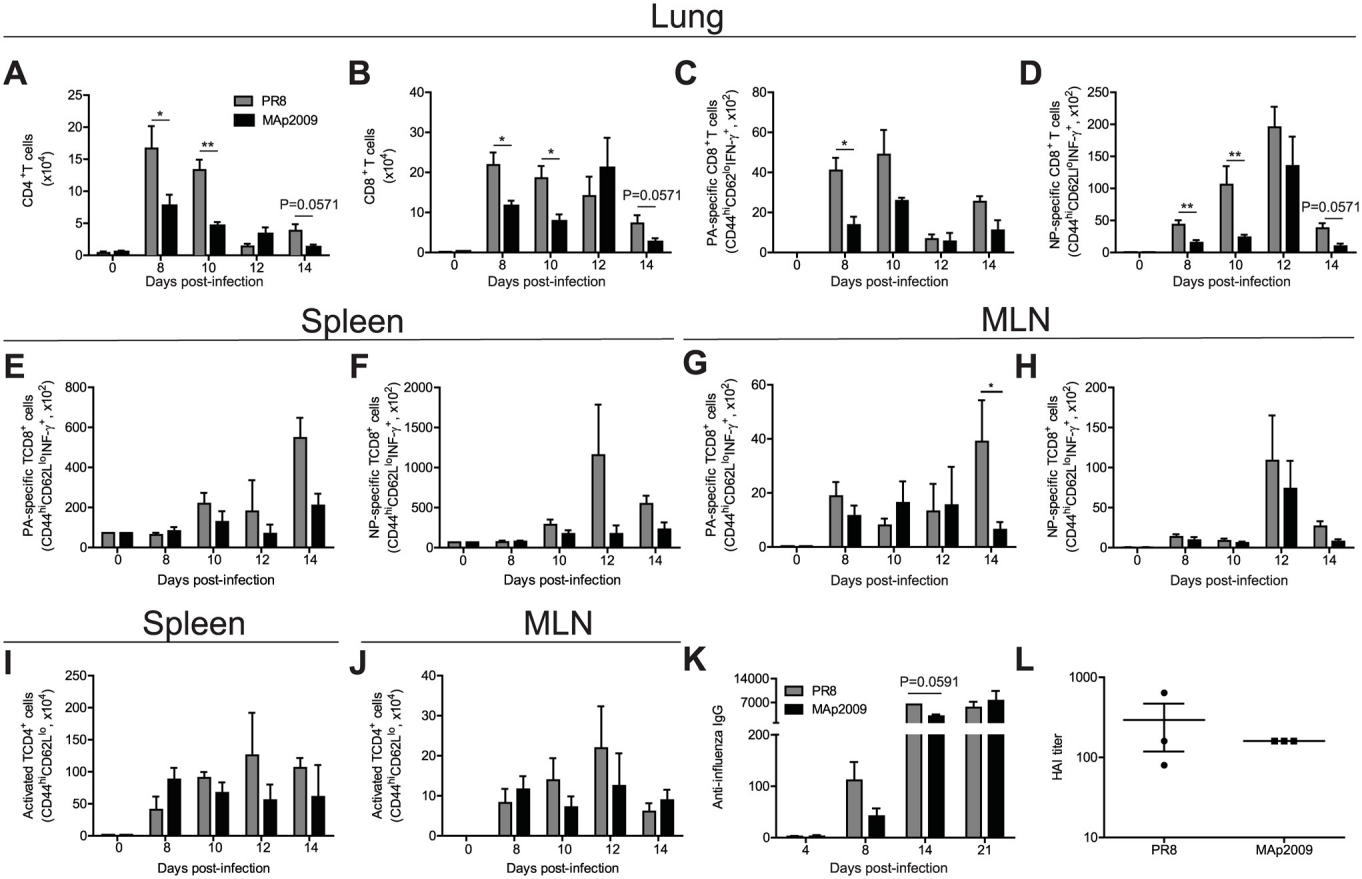
Infection with MAp2009 is associated with an impaired T cell response with little effect on antibody production. Quantification of total CD4^+^ T cells (**A**) and CD8^+^ T cells (**B**) in the lungs of infected mice at the indicated time points. Cells were stimulated with PA (**C**) or NP (**D**) peptides specific for each virus for 6 h in the presence of brefeldin A and the numbers of effector CD8^+^ T cells (CD44^hi^CD62^lo^) producing IFN-γ were quantified by flow cytometry. Total CD4^+^ T cell and CD8^+^ T cell numbers in the spleen (**E**), (**F**), and in the MLN (**G**), (**H**), respectively. Number of activated CD4^+^ T cell in the spleen (**I**) and in the MLN (**J**). (**K**) Total influenza-specific IgG titers. Serum samples were taken at the indicated time points and titers were determined by IPMA. (**L**) Neutralizing antibody production was determined by a HAI assay at day 21 post-infection after pre-incubation of sera with chicken erythrocytes to remove non-specific agglutination activity. Data were analysed using a Mann-Whitney test (*P< 0.05, **P< 0.01) and represent the mean of at least 3 animals from 2–3 independent experiments.

Upon IAV infection, a virus-specific T cell response is triggered and numerous studies have demonstrated its crucial role in the control of infection. Particularly, the contribution of cytotoxic CD8^+^ T cells has been shown to be critical for viral clearance in mice [49,50,51]. Therefore, we next sought to determine whether T cell immunity was affected in the MAp2009-infected mice. Flow cytometric analyses first revealed that the total numbers of CD4^+^ and CD8^+^ T cells were significantly lower in the lungs of animals infected with the MAp2009 virus at days 8 and 10 (*P* < 0.05 at day 8 and *P* < 0.01 at day 10 for CD4^+^ T cells; and *P* < 0.05 for CD8^+^ T cells at day 8 and 10 p.i.), and had a tendency to be lower at day 14 p.i. (Fig 5A and 5B).

In C57BL/6 mice, viral nucleoprotein (NP) and acidic polymerase (PA) are the main targets of the CD8^+^ T cell response [52,53,54]. Virus-specific IFN-γ^+^ effector CD8^+^ T cells were recruited in the lungs of both groups; however, infection with the MAp2009 virus was associated with remarkably weaker NP- and PA-specific CD8^+^ T cell responses (Fig 5C and 5D, *P*<0.05 and *P*< 0.001, respectively) that was maintained over the course of the response (until day 14 p.i.). Given the crucial role of CD8^+^ T cells in viral clearance, these results might explain the prolonged lung viral titers observed previously (Fig 1C). Similar results were observed in the BAL (data not shown). Overall, the numbers virus-specific CD8^+^ T cells were similar in the MLN and in the spleen in MAp2009 and PR8-infected mice in the first 12 days (Fig 5E–5H). At day 14 p.i., a significantly greater number of PA-specific CD8^+^ T cells was found in the MLN of PR8-infected mice (Fig 5G). However, this does not explain the differences in lung viral titers and mouse morbidity observed during the course of infection. Furthermore, the number of activated CD4^+^ T cells in the spleen and in the MLN was similar in both groups (Fig 5I and 5J), suggesting that there is no defect in the generation of the T cell response but rather in the migration and/or survival of these cells in the lungs of MAp2009-infected mice.

Finally, by directly recognizing viral particles at the site of infection, the humoral immune response also contributes to the control of viral replication and dissemination [55]. Therefore, we investigated whether antibody production differed between MAp2009 and PR8-infected animals. First, to monitor the influenza-specific antibody response, we quantified the total antiviral IgG by IPMA in sera harvested at days 4, 8, and 14 p.i. Influenza-specific IgG titers began to be detectable at day 8 after infection and rose importantly at day 14 (Fig 5I). Interestingly, PR8-infected mice had up to 2-fold more IgG at days 8, 14, and similar levels at day 21 p.i. compared to animals infected with MAp2009. However, no major difference was observed in HAI assays using sera collected at day 21(Fig 5J). Thus, the latter results suggest that while T cell responses seemed to be impaired during MAp2009 infection, the humoral immune response was less affected.

### Impaired T cell response is not due to inadequate numbers of dendritic cells in the lungs or in the draining lymph node

**Fig 6:**
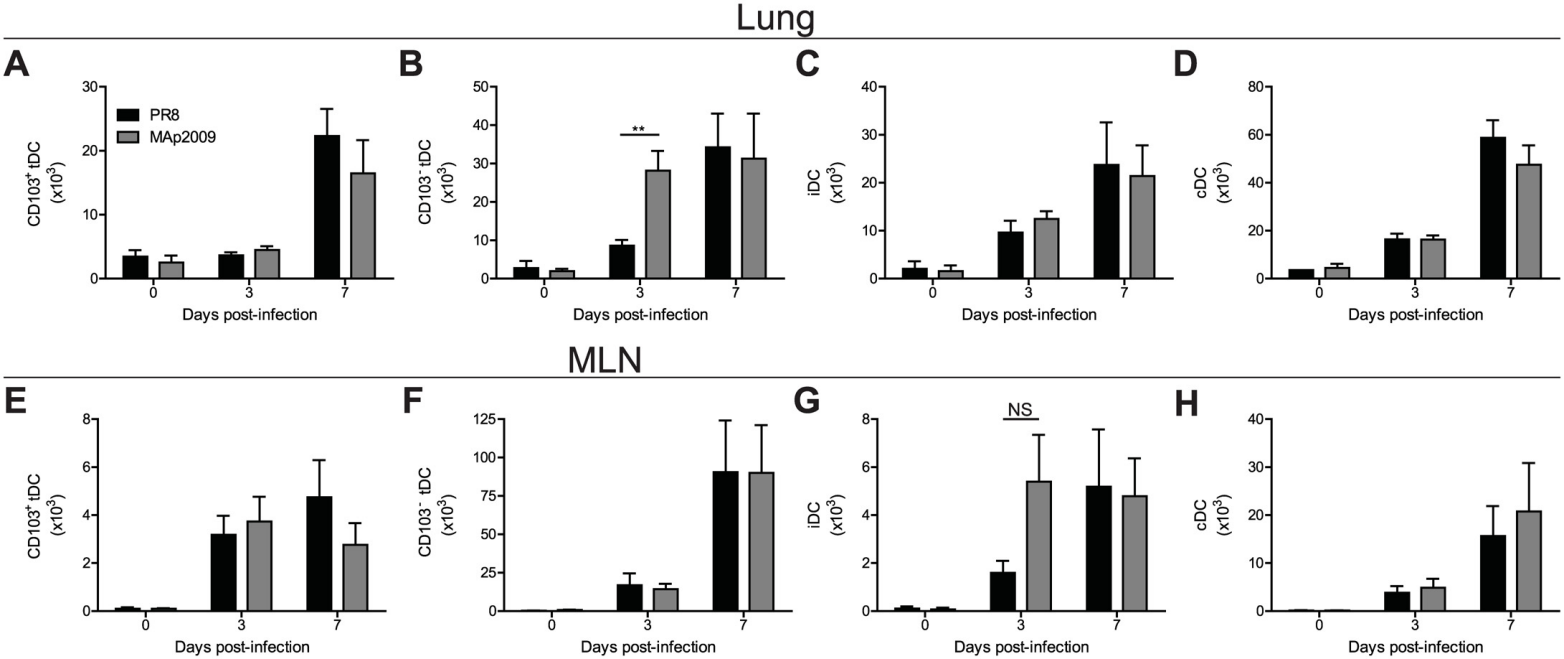
Accumulation of dendritic cells subsets in the lungs and in the draining lymph node does not explain the impaired T cell response observed in MAp2009-infected mice. Quantification of CD103^+^ (**A**) or CD103^-^ (**B**) lung tissue dendritic cells (tDC), inflammatory DC (iDC) (**C**), and conventional DC (cDC) (**D**) at the indicated time points. Cell numbers of CD103^+^ tDC (**E**), CD103^-^ tDC (**F**), and cDC (**G**) in the MLN. Mice were infected with 10 PFU of PR8 or MAp2009 and lungs and MLN were harvested at the indicated time points and cells were analyzed by flow cytometry. tDC were identified as B220^-^MHCII^+^CD11b^+^Ly-6C^lo^ cells, iDC as B220^-^MHCII^+^CD11b^+^Ly-6C^hi^ cells, and cDC as B220^-^MHCII^+^CD11b^+^ CD11c^hi^Ly-6C^lo^. Data were analysed by an unpaired Mann-Whitney test (**P< 0.01) and represent the mean of at least 9 animals from 3–4 independent experiments.

Because DCs are important for the generation of the adaptive immune response through antigen presentation and T cell activation, we sought to determine whether differences in DC subsets might explain the observed impairment of the T cell response in the lungs. Infection with both viruses triggered the accumulation of tDCs. CD103^-^ cell numbers started to increase at day 3 p.i. followed by CD103^+^ cells at day 7 (Fig 6A and 6B). Whereas there was no significant difference in the numbers of CD103^+^ tissue resident DC (tDC) between MAp2009- and PR8-infected mice, we observed significantly more CD103^-^ tDCs at day 3 p.i. in the lungs of mice infected with MAp2009 (*P*< 0.01). iDCs started to increase in the lungs of infected animals after 3 days of infection with either MAp2009 or PR8 and they continued to accumulate at day 7 p.i. (Fig 6C). No difference in inflammatory DC (iDC) cell numbers was observed between both groups. Furthermore, after infection with MAp2009 or PR8, the cDC population began to increase at day 3 p.i. and it continued to rise until day 7 (Fig 6D). Again, no significant difference could be observed between the two groups. Finally, we quantified the same DC subsets in the MLN. The kinetics of DC accumulation in MLN mirrored those found in the lungs (Fig 6E–6H). DCs started to accumulate in the lymph node at day 3 p.i. and numbers continued to rise until day 7. The population found in MLN was generally smaller than that found in the lungs, except for CD103^-^ tDC, especially at day 7 p.i. (Fig 6F). However, no difference was observed between both groups. Therefore, these results suggest that the impaired CD8^+^ T response cannot be attributed to deficient priming due to the lack of DCs in the lungs or deficient migration of these cells into the draining lymph nodes.

### Impaired T cell response is not due to altered chemokine receptor expression

**Fig 7:**
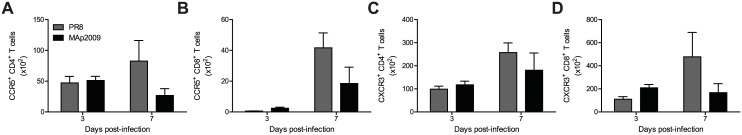
Expression of chemokine receptors involved in T cell migration into the lungs is not impaired in MAp2009 infected animals. Number of CD4^+^ T cells (**A**) or CD8^+^ T cells (**B**) expressing CCR5, and number of CD4^+^ T cells (**C**) or CD8^+^ T cells (**D**) expressing CXCR3. Mice were infected with 10 PFU of PR8 or MAp2009 and lungs were harvested at days 3 and 7 post-infection and the expression of the chemokine receptors CCR5 and CXCR3 on T cells was assessed by flow cytometry. Data were analysed by an unpaired Mann-Whitney test and represent the mean of at least 5 animals from 2 independent experiments.

Once activated in the lymph nodes, T cells need to migrate back to the lungs in order to kill infected cells or to produce cytokines. Therefore, chemokine production as well as expression of the corresponding receptor at the cell surface is crucial. Hence, we analysed the expression CCR5 and CXCR3, two important chemokine receptors for T cell migration at cell surface. CD4^+^ and CD8^+^ T cells expressing CCR5 and CXCR3 were detected as early as 3 days post-infection in the lungs and increased at day 7 (Fig 7). Despite a small trend for higher numbers of CCR5 and CXCR3 positive T cells in the PR8-infected group, no significant differences were detected, suggesting that the observed impaired T cell response is not due to deficient expression of these chemokine receptors.

## Discussion

Influenza viruses generally cause a mild infection of the upper respiratory tract. However, infection with certain strains results in severe disease with viral pneumonia, extensive lung damage, multi-organ failure, and death [10,56,57]. The A(H1N1)pdm09 virus had a fatality rate quite similar to seasonal IAV, ranging from 0.001–0.007% of worldwide population compared to 0.004–0.008% during seasonal disease, respectively. Nevertheless, A(H1N1)pdm09 differed from seasonal IAV as about 64% of fatal cases were in the young and middle-aged population rather than in the very young and elderly [56,57]. Since factors contributing to the high virulence of certain strains of the A(H1N1)pdm09 are still poorly understood, we generated a mouse-adapted A(H1N1)pdm09 strain (MAp2009) and compared its pathogenesis and host response to the laboratory H1N1 PR8 virus. We observed that infection of mice with MAp2009 was associated with increased morbidity, high viral loads and decreased survival. Furthermore, expression of inflammatory cytokines was generally increased in mice infected with the MAp2009 virus. Interestingly, while no statistically significant differences were observed in the numbers of innate immune cells present in the lungs, we showed that total numbers of CD4^+^ T and CD8^+^ T cells and the numbers of virus-specific CD8^+^ T cells were significantly reduced following infection with MAp2009, indicating that virulence of this virus could be attributed to an impaired cytotoxic response.

### Infection of mice with the MAp2009 virus leads to severe disease and high mortality

Human fatal cases of the A(H1N1)pdm09 virus were characterized by high and prolonged viral loads in the nasopharyngeal and endotracheal lavages and infection of the lung parenchyma [9,10,39,58]. Moreover, infection of macaques and ferrets with different isolates of the viruses revealed that the A(H1N1)pdm09 was more virulent than a seasonal strain, with high viral loads, delayed clearance, and lower survival [37,38,59,60]. Here, we observed that, although both viruses being H1N1 strains, at the same initial infectious dose, the MAp2009 virus caused a dramatically more severe disease than the PR8 virus in mice, which was associated with increased weight loss. Strikingly, while all PR8-infected animals survived, MAp2009 infection was associated with 85% mortality. These experiments allowed us to determine that the MLD_50_ of MAp2009 was 7 PFU, while that of PR8 is 100 PFU. Finally, in accordance with previous studies, we observed that infection with MAp2009 was associated with higher viral loads and delayed viral clearance in comparison with the PR8 virus. These results demonstrate that the mouse-adapted virus derived from an isolate of the A(H1N1)pdm09 is more virulent than the commonly used laboratory strain PR8, which is in accordance with previously observed results in other animal models.

### The MAp2009 virus influences the cytokine profile in the lungs of infected mice but has little effect on the accumulation of innate immune cells

Upon entry of the virus by endocytosis in airway epithelial cells, viral infection can be detected through many innate sensors such as Toll-like receptors (TLR) [61,62], retinoic acid-induced like receptor (RIG-I), melanoma-differentiation gene 5 (MDA5) and Nod-like receptor 2 (NOD2). Activation of these receptors leads to the rapid secretion of type I IFN, activation of ISG, but also induces inflammatory responses, such as the activation of the inflammasome, production of many NF-κB-dependent inflammatory cytokines, and secretion of chemokine, which allows the establishment of an antiviral state in infected as well as in the neighboring cells [63,64,65,66]. Severe IAV infections, such as those caused by some H5N1 viruses and the A(H1N1)pdm09, were characterized by excessive production of inflammatory cytokines [36,40,67,68,69]. Consistent with these studies, we observed that mice infected with the MAp2009 virus had generally stronger expression of IFNs, ISGs and cytokines compared to the PR8-infected group. This difference correlated well with the higher viral loads in mice infected with MAp2009. Chemokines produced by epithelial cells and resident innate immune cells control leukocyte migration to the airways [70,71]. Although not statistically significant, we did observe a tendency in the MAp2009-infected animals to have higher numbers of innate immune cells at day 7 p.i. Further studies of cellular activation/migration or of the expression of adhesion molecules on epithelial cells, such as ICAM-1 and VCAM-1 [72], which are essential for transmigration of innate cells, could provide further insight to understand why the difference in the number of innate immune cells is not more striking.

### Infection with the more virulent MAp2009 results in an impaired T cell response

CD4^+^ T cells and CD8^+^ T cells both have important but distinct roles in the control of IAV replication and clearance. While CD4^+^ T cells are thought to act mainly by providing secondary signals for the humoral immune response through the production of cytokines, CD8^+^ T cells limit viral spread by eliminating infected cells [73,74,75]. Interestingly, patients that developed severe disease following A(H1N1)pdm09 infection were found to have fewer CD4^+^ T cells, CD8^+^ T cells and B cells in the serum and have lower expression of genes involved in the adaptive immunity such as in T and B cell signaling, CD4^+^ T cell differentiation, and in B cell development, suggesting an impaired adaptive immune response [8,34,76]. Here, we observed that the total numbers of CD4^+^ T cells and CD8^+^ T cells in the lungs following MAp2009 infection were significantly lower at peak of the T cell response (days 8 and 10) compared to the PR8-infected group. More importantly, MAp2009-infected mice had significantly fewer virus-specific effectors CD8^+^ T cells at the site of infection, suggesting that they also have an impaired adaptive immune response. The lack of a prompt and efficient T cell response following infection with the MAp2009 virus [73,74,75], which is crucial for the control of viral replication, therefore results in higher viral loads, increased disease severity, and death. Despite the significantly lower numbers of CD4^+^ T cells, we observed a small effect on the total influenza-specific IgG titers, while there was no difference in the neutralizing response. Overall, these results are in accordance with human studies that demonstrated that severe cases of A(H1N1)pdm09 are associated with an impaired T cell response.

### Why is the T cell response impaired following infection with MAp2009?

Viruses have developed many strategies to interfere with the adaptive immune response. The human immunodeficiency virus (HIV) and hepatitis B and C viruses (HBV and HCV) upregulate the expression of programmed death protein 1 (PD-1) at the surface of CD8^+^ T cells to induce their exhaustion [77,78]. Furthermore, rabies virus destroys T cells by inducing the overexpression of immunosubversive molecules, such as FasL, HLA-G or B7-H1 [79]. In our studies, MAp2009-infected animals did not have more PD-L1-expressing cells and they did not have more apoptotic T cells in the lungs (data not shown). Furthermore, the number of CD4^+^ T cells and virus-specific CD8^+^ T cells in the MLN and in the spleen did not differ between MAp2009 and PR8-infected mice, suggesting that the generation of the T cell response is not affected. Therefore, these data suggest that there might be a defect in the processes involving migration of the newly generated virus-specific T cells to the site of infection.

Following IAV infection, DCs capture antigens and migrate to the MLN. Therein, they encounter naïve CD4^+^ T and CD8 T ^+^ cells that will undergo a process of activation, proliferation, and differentiation to become effector cells [80,81]. Subsequently, these cells will migrate back into the airways to eliminate infected cells. Lung resident DCs are important to provide continuous signals to guide further activation and effector functions [82,83,84]. To determine if the observed impaired T cell response is a consequence of the lack of DC in the airways or in the MLN, we quantified various subsets of DCs known to be involved in antigen presentation and in cytokine production [81,85,86]. Tissue resident DC (tDC) can be subdivided based on the expression of the α_E_β_7_ integrin (CD103). Both CD103^+^ and CD103^-^ tDCs are found in the lungs and migrate to MLN after IAV inflection. However, tDCs CD103^-^ are considered as more potent antigen presenting cells [87]. Despite the differences in the T cell response observed after infection with PR8 or MAp2009, no significant differences in the numbers of tDCs were detected. Monocyte-derived DCs (iDCs) are known to produce large amounts of cytokines and have been shown to be recruited to the MLN after IAV infection [86,88]. Again, mice infected with either PR8 or MAp2009 exhibited similar numbers of these cells. Finally, cDCs are known to acquire antigen in the lungs and migrate to MLN and are often implicated as the major subset for cross-priming CD8^+^ T cells [89,90,91,92]. As with the other DC subsets, no difference significant differences in cDC numbers were observed after PR8 or MAp2009 infection. These results therefore suggest that the impaired CD8^+^ T response is not caused by deficient priming due to the lack of DCs in the lungs or in the draining lymph nodes.

After infection, DCs bearing viral antigen migrate to the lymph node where they activate naïve virus-specific T cells. These cells proliferate, leave the lymph node, and enter into the bloodstream to enter into the lungs by extravasation [93,94,95,96]. This migration process is tightly regulated by the production of diverse chemokine such as CCL5 and CXCL10 and recognition by their respective receptor CCR5 and CXCR3 [97,98]. Despite the impaired T cell response observed in MAp2009-infected mice, we did not detect any altered expression of these chemokine receptors. Therefore, the lower T cell response cannot be explained by lack of chemokine receptor expression. More in-depth studies on T cell migration could shed light on the mechanisms leading to impairment of the T cell response following MAp2009 infection.

While further investigation is needed to fully understand the underlying mechanisms, our findings suggest that an inefficient T cell response leads to inefficient control of viral replication, increased lung pathology, morbidity and survival in MAp2009-infected mice.
